# Size Distribution Imaging by Non-Uniform Oscillating-Gradient Spin Echo (NOGSE) MRI

**DOI:** 10.1371/journal.pone.0133201

**Published:** 2015-07-21

**Authors:** Noam Shemesh, Gonzalo A. Álvarez, Lucio Frydman

**Affiliations:** Department of Chemical Physics, Weizmann Institute of Science, Rehovot, 76100, Israel; National Institute of Radiological Sciences, JAPAN

## Abstract

Objects making up complex porous systems in Nature usually span a range of sizes. These size distributions play fundamental roles in defining the physicochemical, biophysical and physiological properties of a wide variety of systems – ranging from advanced catalytic materials to Central Nervous System diseases. Accurate and noninvasive measurements of size distributions in opaque, three-dimensional objects, have thus remained long-standing and important challenges. Herein we describe how a recently introduced diffusion-based magnetic resonance methodology, Non-Uniform-Oscillating-Gradient-Spin-Echo (NOGSE), can determine such distributions noninvasively. The method relies on its ability to probe confining lengths with a (*length*)^6^ parametric sensitivity, in a constant-time, constant-number-of-gradients fashion; combined, these attributes provide sufficient sensitivity for characterizing the underlying distributions in μm-scaled cellular systems. Theoretical derivations and simulations are presented to verify NOGSE’s ability to faithfully reconstruct size distributions through suitable modeling of their distribution parameters. Experiments in yeast cell suspensions – where the ground truth can be determined from ancillary microscopy – corroborate these trends experimentally. Finally, by appending to the NOGSE protocol an imaging acquisition, novel MRI maps of cellular size distributions were collected from a mouse brain. The ensuing micro-architectural contrasts successfully delineated distinctive hallmark anatomical sub-structures, in both white matter and gray matter tissues, in a non-invasive manner. Such findings highlight NOGSE’s potential for characterizing aberrations in cellular size distributions upon disease, or during normal processes such as development.

## Introduction

Cellular morphologies are intimately linked with biological functions in general, and with a tissue’s capacity to perform its physiological role *in-vivo* in particular. Cell sizes can determine, *inter-alia*, the Central-Nervous-System (CNS) axonal conduction velocities [1], and the efficiency of oxygen transport by red-blood cells [2]. When viewed as an ensemble, cellular sizes are nearly invariably dispersed across a distribution, whose profile is tightly regulated by elaborate mechanisms aiming to maintain the optimal cellular dimensions for a proper physiological function [3]. In mammalian brains, for instance, distinct cortical regions evolved slight–but functionally crucial [4]–variations in their neuronal size distributions, which enable the execution of different kinds of neural computations [5]. Furthermore, even slight aberrations in axonal sizes have been found related to severe neurological disorders [6–8]. Most evidence about the mutual dependencies between such physiological processes and underlying size-dependent distributions, arises from *ex-vivo* histological studies. Clearly, having the capability to image cellular size distributions non-invasively and under *in-vivo* conditions, could be crucial for understanding the subtle but important connections between morphological features and normal processes like maturation [9] or plasticity [10], as well as for predicting and understanding the nature of CNS diseases [11].

Owing to its noninvasiveness and multi-modal contrasts, Magnetic Resonance Imaging (MRI) has evolved into a central technique for *in-vivo* investigations of bulk structural, functional and metabolic aspects of the CNS [12]. Diffusion-based MRI in particular, has been used to shed light on structures whose dimensions are orders-of-magnitude smaller than the imaged voxel sizes [13,14]. This is made possible by detecting restricted micron-scale Brownian motions undergone by endogenous water molecules, diffusing within the tissue’s micro-architecture. Such restriction-related phenomena have proven instrumental in the early diagnosis of ischemia [15], as well as for mapping white matter (WM) connectivity [13]. MRI methods for the robust mapping of *in-vivo* size distributions, however, remain elusive. So-called *q-*space imaging [16,17] can generate exquisite contrasts reflecting regional average axonal diameters [18]. Still, quantifying key parameters of such size distributions (e.g., their mode, peak and width) remains subject to elaborate assumptions on the nature of the tissues involved, while requiring the application of extremely strong gradients for probing small compartment dimensions [19–21]. Related microstructure-probing methods have also been put forward [22–24], but these likewise require complex analyses for parameterizing the distributions, while also requiring the application of very strong gradients [25]. These difficulties reflect *q-*space’s limited parametric sensitivity towards the average length *l*_*c*_ defining the confinement, which in these approaches varies as $l_{c}^{2}$. An alternative approach enhancing MRI’s sensitivity towards small compartment sizes relies on utilizing oscillating gradient waveforms [26–30]. In particular, the $l_{c}^{4}$ parametric sensitivity of Oscillating-Gradient Spin-Echo (OGSE) MRI towards the confinement lengths by determining a diffusion spectrum, enables one to probe small elements in the size distribution by suitably tailoring the gradient’s waveform [29,31,32].

The OGSE approach is designed to scan a diffusion spectrum, and from its width one can extract the restriction length *l*_*c*_ [30]. Alternatively, if the functional form of the diffusion spectrum is known, one could design an OGSE-like sequence to directly extract the restriction length. This study explores the potential for mapping subtle features of compartment size distributions in this manner, using a recently introduced methodology that probes confining lengths with a $l_{c}^{6}$ parametric sensitivity [33,34]. At the core of our approach lies a Selective-Dynamical-Recoupling (SDR) variant known as Non-uniform-Oscillating-Gradient Spin-Echo (NOGSE), which in previous studies was shown to accurately extract monodisperse or average pore sizes in a constant-time, constant-number-of-gradients fashion [35]. The constant-time, constant-number-of-gradients features of this family of sequences allows one to factor out T_2_ and gradient-switching related weightings from the signal. The present study investigates how this microstructural $l_{c}^{6}$ parametric sensitivity can be further exploited to probe the parameters of cell-scale size distributions in the 1–10 μm range. To this effect we discuss first the method’s principles, including simulations demonstrating NOGSE’s ability to report on size distributions. The methodology is then validated in yeast cells suspensions, where excellent matching is obtained against a ground-truth stemming from optical microscopy. Finally, NOGSE is combined with MRI measurements to map the salient features of size distributions in mouse brains. These measurements clearly reveal hallmark microstructural features of the CNS, in both white and gray matter tissues. The prospects of exploiting the ensuing size distribution contrasts for correlating between μm-size morphologies and CNS maturation and disorders in human-oriented settings, are discussed.

## Theoretical Background: Size Distributions from NOGSE Measurements

**Fig 1:**
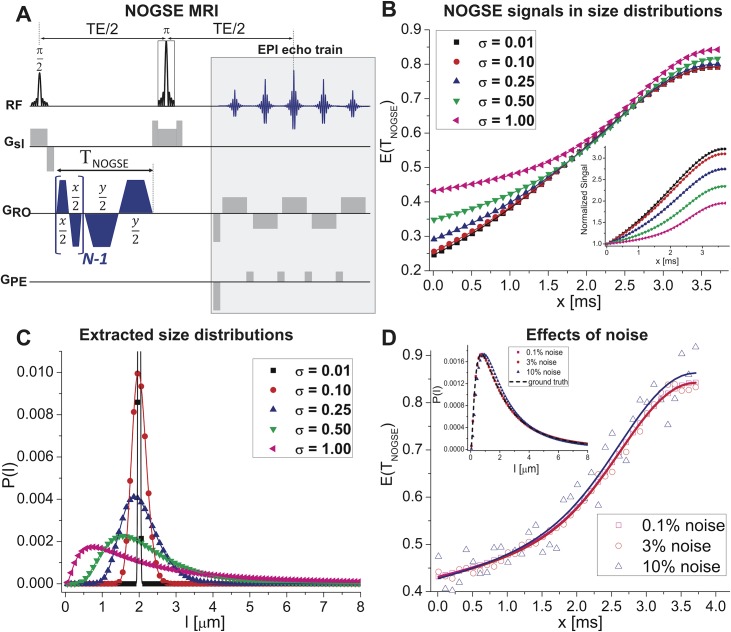
Characterizing size distributions from NOGSE data. **(A)** NOGSE MRI sequence used, encompassing an initial block probing the confinements over a time T_NOGSE_, and a single-shot spin-echo Echo-Planar-Imaging readout (NOGSE gradients are shown along the RO direction, but can be applied in arbitrary orientations). **(B)** *x* time-dependence of the NOGSE signal attenuation *E(T*_*NOGSE*_ *)* for different size distributions. Note that as the lognormal distribution width increases the *E(T*_*NOGSE*_ *)* changes both in curvature and in overall amplitude; the inset highlights this by normalizing the curves to their first point (Min(*x*)). **(C)** Probability distributions *P(l)* extracted from fitting the simulations in (B) for a given restricting length *l* in a noise-less reconstruction. The extracted distributions overlap perfectly with the simulated ones. **(D)** Effects of adding noise to the NOGSE signal for the widest distribution considered in (C): notice that even when fluctuations reach 10% of the signal, the fits remain robust and the distributions are well reconstructed (inset). Throughout this Fig symbols represent the synthetic data whereas solid curves represent fits to these data. For all distributions *l*_*c*_ = 2 μm, *G* = 40 G/cm, *N* = 8, *T*_*NOGSE*_ = 30ms.

SDR is a recently-developed methodology [33] which utilizes dynamical decoupling concepts [36] to characterize frequency fluctuations in a constant-time fashion, via non-equidistant multiple π-pulse echo trains. When used to monitor diffusion-driven fluctuations, SDR offers a simple way for quantifying confinements with a $l_{c}^{6}$ parametric sensitivity by microscopically characterizing the diffusion process, rather than by fitting apparent-diffusion weightings/decays [34]. This is performed by systematically changing a single time-delay variable in a constant time fashion and with a fixed number of refocusing π-pulses, which provides robustness against progressive T_2_ decay and cumulative refocusing pulse errors, respectively. In the context of compartment size estimations, the opportunity arises of replacing SDR’s multi-pulse echo trains by a gradient waveform modulation [34,35]. The resulting Non-uniform-Oscillating-Gradient-Spin-Echo (NOGSE) methodology involves *(N-1)* gradient oscillations akin to those in a Carr-Purcell-Meiboom-Gill (CPMG) like modulation characterized by a variable time *x* [37], followed by a single Hahn-echo-like oscillation of period *y* such that (*N* − 1) * *x* + *y* ≡ *T*_*NOGSE*_ –where *T*_*NOGSE*_ is a constant (Fig 1A). The diffusion-weighted signal attenuation *E*(*T*_*NOGSE*_) arising from a spin ensemble within a single compartment that is dephased by this oscillating gradient waveform ***G*** *(t)*, can be described as *M*_*NOGSE*_(*T*_*NOGSE*_) = exp{−*β*(*T*_*NOGSE*_)}. Here the attenuation factor $\beta \left(T_{NOGSE}\right)=\frac{1}{2}\int_{-\infty }^{\infty }{\left|F\left(\omega ,T_{NOGSE}\right)\right|}^{2}S\left(\omega \right)d\omega$ [30,30,34,35,37], where *S(ω)* is a spectral density given by the Fourier Transform (FT) of the spins’ displacement correlation function $g\left(\tau \right)/\sqrt{2\pi }=\langle r\left(0\right)r\left(\tau \right)\rangle /\sqrt{2\pi },$ and *F*(*ω*, *T*_*NOGSE*_) is a filter function given by the FT of the gradient modulation $\sqrt{2\pi }\gamma G\left(t\right)$. Under typical restricted diffusion conditions the spectral density will be given by $S\left(\omega \right)=\frac{D_{0}\tau_{c}^{2}}{\left(1+\omega^{2}\tau_{c}^{2}\right)\pi }$ [34], where *τ*_*c*_ is the time required for most molecules to fully probe the pore boundaries [34,38,39]. *τ*_*c*_ is therefore related to the confining length scale *l*_*c*_ by the Einstein-Smoluchowski expression $l_{c}^{2}=2D_{0}\tau_{c},$ where *D*_*0*_ is the free diffusion coefficient. For more complex geometries the displacement power spectrum *S*(*ω*) can be written as a sum of Lorentzian terms [35,39]; however, given that for typical geometries (cylinder, spheres, planar layers) the contribution from the second term in these series expansions is lower than 2% [39], we shall for simplicity ignore all but the dominant term in this study.

NOGSE’s ability to probe confinements as *l*_*c*_^*6*^, derives from the signal amplitude modulation that it exhibits as a function of the delay *x*. This modulation will vary between values corresponding to a pure *x* = 0 Hahn-echo oscillation, *M*_*NOGSE*_(*T*_*NOGSE*_, *N*, *x* = 0) = *M*_*Hahn*_(*T*_*NOGSE*_), and values arising from a CPMG gradient oscillation where *x* = *T*_NOGSE_/*N*: *M*_*NOGSE*_(*T*_*NOGSE*_, *N*, *x* = *T*_*NOGSE*_/*N*) = *M*_*CPMG*_(*T*_*NOGSE*_, *N*). The difference between these two limiting values represents NOGSE’s amplitude modulation, *ΔM*_*NOGSE*_ = *M*_*CPMG*_(*T*_*NOGSE*_, *N*) − *M*_*Hahn*_(*T*_*NOGSE*_) ∝ exp[−*Δβ*_*NOGSE*_(*T*_*NOGSE*_, *N*)] − 1, whose value can then be monitored as a function of *x*. If *T*_NOGSE_/*N*>>*τ*_*c*_, *i*.*e*. if the diffusing spins experience a restriction imposed by a confining topology, the CPMG-related attenuation factor $\beta_{CPMG}\left(T_{NOGSE},N\right)∼\gamma^{2}G^{2}D_{0}\tau_{c}^{2}\left[T_{NOGSE}-\left(2N+1\right)\tau_{c}\right]$ [34]. This includes a dominant term $\propto \tau_{c}^{2},$ well-known from OGSE-type experiments [38,39], plus a correction proportional to $\tau_{c}^{3}\propto l_{c}^{6}.$ The Hahn-related attenuation factor (*N* = 1) under similar conditions is given by $\beta_{Hahn}\left(T_{NOGSE},N\right)∼\gamma^{2}G^{2}D_{0}\tau_{c}^{2}\left[T_{NOGSE}-3\tau_{c}\right]$. By combining these two elements in a single pulse sequence, NOGSE delivers a modulation depending on the *difference* between these attenuation factors: $\Delta \beta_{NOGSE}^{restricted}\;\left(T_{NOGSE},N\right)=-\frac{1}{4D_{0}^{2}}\;{\left(\gamma G\right)}^{2}\;*\;\left(N-1\right)\;l_{c}^{6}$. This explains the experiment’s *l*_*c*_^*6*^ dependence on restriction lengths, which is different from either *q*-space’s quadratic or OGSE’s quartic conventional dependencies. Notice that whereas OGSE experiments are mostly analyzed in the frequency domain in order to discriminate the transition from free to restricted diffusion regimes [27,28,30], NOGSE is performed and analyzed in the time domain as a function of the aforementioned *x* parameter. This reveals the transition between free and restricted diffusion regimes as an amplitude modulation, mimicking the time evolution of the mean square root displacement of the diffusing spins [34,35]. By virtue of the sixth-power law of NOGSE’s amplitude modulation, one could envision a subset of experimental conditions and substrates (μm sizes, suitable relaxation delays, moderate gradients) whereby NOGSE could have a good ability to distinguish different restriction lengths; examining whether this potential translates into a useful new tool to characterize size distributions, is the main goal of this work.

To do so we assume that the NMR signal in most porous system can be considered as arising from an ensemble of compartments with potentially different sizes *l*. The total signal modulation in these experiments will thus be given by *S*_*NOGSE*_(*x*) = ∑_*l*_ *P*(*l*)*M*_*NOGSE*_^*l*^(*x*), where *P*(*l*) is the compartment size’s probability distribution characterizing the ensemble, and *M*_*NOGSE*_(*x*) is as described above. As a model for the compartment size’s distribution we chose a lognormal distribution of the form $P\left(l\right)=\frac{1}{l*ln\left(\sigma \right)\sqrt{2\pi }}e^{-\frac{{\left(\text{ln}\left(l\right)-\text{ln}\left(l_{c}\right)\right)}^{2}}{2\text{ln}{\left(\sigma \right)}^{2}}}$, where *l*_*c*_ is the mean compartment size and *σ* is the width of the probability distribution about its mean. Although a different distribution could be clearly chosen, we preferred this specific distribution as it often describes well CNS-type systems [40,41]; as further illustrated below, experimental results were consistent with this model. Notice as well that given the assumed functional for *S*(*ω*), the pores being characterized are considered completely hermetic and described by an average restriction length. Therefore, certain effects such as incomplete restrictions, exchange between compartments or permeability cases, will not be optimally described by our model. These effects will still be accounted for, either by a larger effective pore size, or by a change in the distribution of ensemble sizes. To account for such complexities in a more accurate fashion, further refinements of the Lorentzian *S*(*ω*) model would be necessary.

Before describing the results of these tests it is worth stressing that, if a given signal decay could be unambiguously traced to the effects of diffusion during the application of a perfectly characterized gradient waveform modulation, then *l*_*c*_ could be probed with similar parametric sensitivities using suitable Hahn-based, or CPMG/OGSE-based sequences. In such cases, however, this would require comparing signals arising from measurements that involve different total evolution times or different number of gradient oscillations; under such conditions, T_2_ as well as oscillating gradient waveform imperfections (or cross-terms with background gradients) might introduce attenuation artifacts that could eclipse the pure diffusion signal attenuation. Hence the potential of NOGSE for probing constrained diffusion could be summarized as stemming from: i) the fact that in a favorable *T*_NOGSE_/*N*>>*τ*_*c*_ regime NOGSE’s amplitude modulation will vary as $l_{c}^{6}$, a parametric dependence which might be desirable for microstructural characterizations, and ii) NOGSE $l_{c}^{6}–$modulations are retrieved from a single constant-time, constant-number-of-gradient-modulation sequence, rendering these measurements independent of T_2_ and of gradient-switching related artifacts. In this regard, it is worth pointing out that a higher parametric sensitivity will not always provide a more accurate determination of restriction sizes: a proper tradeoff between the signal attenuation of a diffusion-based experiment and its parametric sensitivity to the sizes being measured, needs to be achieved.

## Materials and Methods

### Simulations

All simulations were performed using Matlab (Mathworks, Natick, MA, USA). The full analytical expressions expected for NOGSE signals in homogeneously confined geometries were recently derived [34,35]. To extend these NOGSE responses to a given distribution of confinements, an array of individual NOGSE signals *M*_*l*_^*NOGSE*^ (*x*) arising from 1989 compartment sizes *l* equidistantly dispersed between 0.056 and 10 μm was first simulated. An intrinsic diffusion coefficient of *D*_*0*_ *=* 0.7x10^-5^ cm^2^/sec was used for generation of these signals, which were subsequently weighted by their fraction in the lognormal distribution, and summed as *S*_*NOGSE*_(*x*) = ∑_*l*_ *P*(*l*)*M*_*l*_^*NOGSE*^ (*x*). Additional specific parameters for the simulations are given in the figure captions.

### Specimen preparation

All experiments were approved by the Institutional Animal Care and Use Committee of the Weizmann Institute of Science under protocol number 10790514–1. Fresh *saccharomyces cerevisiae* Baker’s yeast cells were dissolved in PBS in a 10 mm NMR tube, and left for ~72 hours prior to their MR investigation. Two mice were sacrificed by isoflurane overdose​ and their brains were fixed in formaline, and washed twice with PBS prior to their insertion to a 10 mm NMR tube filled with Fluorinert (Sigma-Aldrich, Rehovot, Israel). All specimens were left in the magnet for at least three hours prior to experiment commencement, to thermally equilibrate.

### MRI experiments

Experiments were performed on a 9.4 T Bruker Avance III equipped with a Micro5 probe capable of producing gradients up to 291 G/cm in all three dimensions. Temperatures were stabilized in the 20–25°C range, and experiments were performed using the NOGSE MRI sequence shown in Fig 1A. For the yeast cells, the following imaging parameters were used: *TR*/*TE* = 4000 / 64 ms, Field of View FOV = 19 x 19 mm^2^ with a matrix size of 64x64, leading to an in-plane resolution of 296x296 (μm)^2^, and slice thickness = 3000 μm and 48 signal averages (total experiment time, ~1.5 hours for the entire curve). NOGSE parameters were *G* = 87 G/cm, *T*_NOGSE_ = 30 ms, *N* = 8, and *x* was varied between 1 and 3.75ms in 29 steps. The mouse brain imaging parameters were as follows: for the sagittal orientation, *TR*/*TE* = 4000 / 100 ms, FOV = 13 x 13 mm^2^ with a matrix size of 144x144, leading to an in-plane resolution of 90x90 (μm)^2^, and slice thickness = 400 μm and 160 signal averages; for the coronal orientation, *TR*/*TE* = 4000 / 91 ms, FOV = 16 x 12 mm^2^ with a matrix size of 192x144, leading to an in-plane resolution of 83x83 (μm)^2^, and slice thickness = 600 μm and 160 signal averages (total experiment time ~5.5 hours for the entire curve). NOGSE parameters for both coronal and sagittal planes were *G* = 57.6 G/cm, *T*_NOGSE_ = 30 ms, *N* = 8, and *x* varied between 0.8 and 3.75 ms in 31 steps. In the corpus callosum experiments, the NOGSE gradients were applied perpendicular to the main axis of the axons; in the coronal experiments, NOGSE experiments were applied in the R-L direction.

### Data analysis

**Fig 2:**
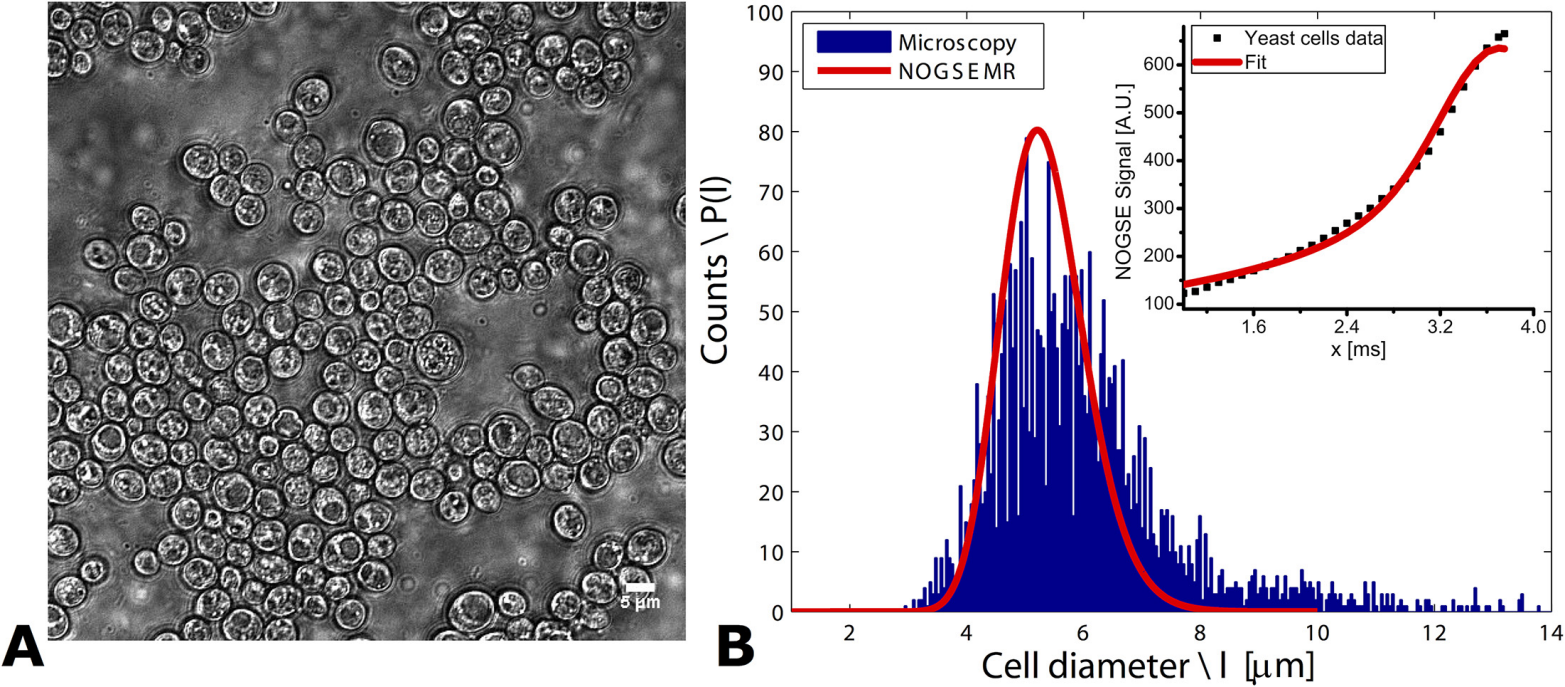
Validating NOGSE’s size distribution predictions in yeast cells. **(A)** A representative image of the examined yeast ensemble; note that objects larger than ~8 μm are not observed in these images, suggesting that the wide right shoulder of the microscopy-derived distribution in (B) arises from unresolved, adjacent cells. **(B)** Size distribution reconstructed from a NOGSE experiment on the yeast ensemble (red curve, with symbols in the inset presenting the experimental data and the solid line their best fit), overlaid on the cellular size distribution obtained from optical microscopy (bin size = ~ 0.05 μm). NOGSE parameters: *T*_*NOGSE*_ = 30 ms, *G* = 87.3 G/cm, *N* = 8. *D*_*0*_ was assumed 1x10^-5^ cm^2^/sec, as this gave the best fits to the data.

All data were analyzed using a home-written code employing Matlab’s lsqnonlin function. When regions-of-interest (ROI) were considered, the mean signal from the pixels in the ROI was analyzed. Maps of the mean, peak value and width of the distribution, were generated from a pixel-by-pixel fit of the experimental data to NOGSE’s theoretical signal decay in the presence of distributions assuming a uniform *D*_*0*_ of 0.7x10^-5^ cm^2^/sec and 1.0x10^-5^ cm^2^/sec in the brains and yeast cells, respectively. The size distributions were fitted by first generating NOGSE signals for an array of restricting lengths ranging between 0.056 and 10 μm, in equidistant 0.005 μm steps. The experimental data was then regressed onto these curves. To avoid local minima, each search began with 18 combinations of different mean and width distributions, and complex-valued solutions were excluded. The best fit was then selected and its distributions parameters were stored. No assumptions on tissue models (e.g., intra/extra-cellular compartments) were made; a single lognormal distribution was thus fitted for each fitted element, regardless of the element’s potential heterogeneity. This implicitly means that the constrained diffusion signal contributions arising from all underlying compartments (e.g., extracellular, intracellular, etc.), are assumed described by a single lognormal distribution weighting. The yeast cells geometry was assumed to be spherical (as was validated by the microscopy results shown in Fig 2B), such that the lognormal distribution described spheres rather than one dimensional objects. A correction factor of 1/0.3 was thus applied to the distribution mean, to account for this spherical compartment shape [30,35]. In the brains, we did not assume any particular geometry, and simply considered a distribution of correlation lengths *l*_*c*_.

### Yeast Cells Microscopy

A sample of the yeast cells was taken directly from the NMR tube and imaged via a DeltaVision system consisting of an Olympus IX71 wide-field inverted fluorescence microscope, an Olympus UPlanApo 63x and a NA 1.40 oil immersion objective (Applied Precision, Seattle, WA, USA). Thirty images containing thousands of yeast cells were subsequently imported in ImageJ, and their Feret diameter (i.e., the longest distance within the oval shape) was automatically quantified. Objects smaller than 2 μm were misidentified by the software recognition algorithm and were hence discarded from the analysis.

## Results

### Validating NOGSE’s ability to extract size distributions

As a test of NOGSE’s ability to extract simple parameters to characterize size distributions–including their mean, peak and widths– signals were first simulated for five lognormal distributions *P(l)* distributed around a biologically-relevant size of *l*_*c*_ = 2 μm, and possessing different distribution widths *σ* (Fig 1B; see *Materials and Methods*for details). Clearly, even small differences *σ* ≈ 0.1^.^ *l*_*c*_ imprint a marked dependence on the amplitude modulations and on the curvatures of the NOGSE signals (inset, Fig 1B). Excellent correspondence was observed when synthetic NOGSE data are given as input, and the originating size distributions are recovered by fitting (Fig 1C). Moreover, although it might be expected that the presence of noise in realistic biological data would influence these fittings, even adding 10% fluctuations (a value that is much higher than the typical noise levels present in typical experiments) yields only marginal variations in the extracted size distributions: all the general features of the distribution including mean, peak and width values, are still captured (Fig 1D, inset). Furthermore, such features are not limited to the particular length we have chosen; supplementary S1 Fig shows further analyses for smaller and larger sizes revealing that, in each case, fits of the NOGSE experiment recapitulate well the underlying size distributions.

With these simulations as background, size distributions were experimentally quantified in a biological system whose ground-truth was determined by an independent modality. Yeast cells were chosen due to their simple spherical geometry, and the NOGSE MRI characterization was checked against a large number (thousands) of cells whose sizes were quantified *ex vivo* by optical microscopy. Results of these measurements are shown in Fig 2; Fig 2B in particular compares the size distributions extracted by fitting experimental NOGSE data (inset), against the cell size histogram obtained by light microscopy. The two size distributions closely resemble one another and peak at the same cell diameter of ~5.8 μm; this value is in close agreement with the average yeast cell size measured via a different diffusion MR methodology [42]. Importantly, not only the maxima but also the widths of the two distributions overlap significantly. Notice that although the microscopy suggests a broadening of the distribution biased towards larger cell sizes, closer inspection of the yeast cells’ images (Fig 2A) evidences that almost no cell is actually larger than 8 μm. The microscopy’s high-*l*_*c*_ tail therefore likely results from artifacts in the image recognition algorithm, and reflects adjacent cells that were not sufficiently resolved in the image to be recognized as separate entities. Minor deviations can also suggest that a different distribution model could be appropriate for characterizing this specimen; in particular, consideration of different models for the intra- and extra-cell signals contributions could perhaps enhance these fits even further. Additional effects due that were not considered in our model–for instance spin exchanges between intra- and extra-cellular compartments– could also contribute to these deviations.

### Mapping cellular size distributions: Non-invasive NOGSE-based brain characterizations

**Fig 3:**
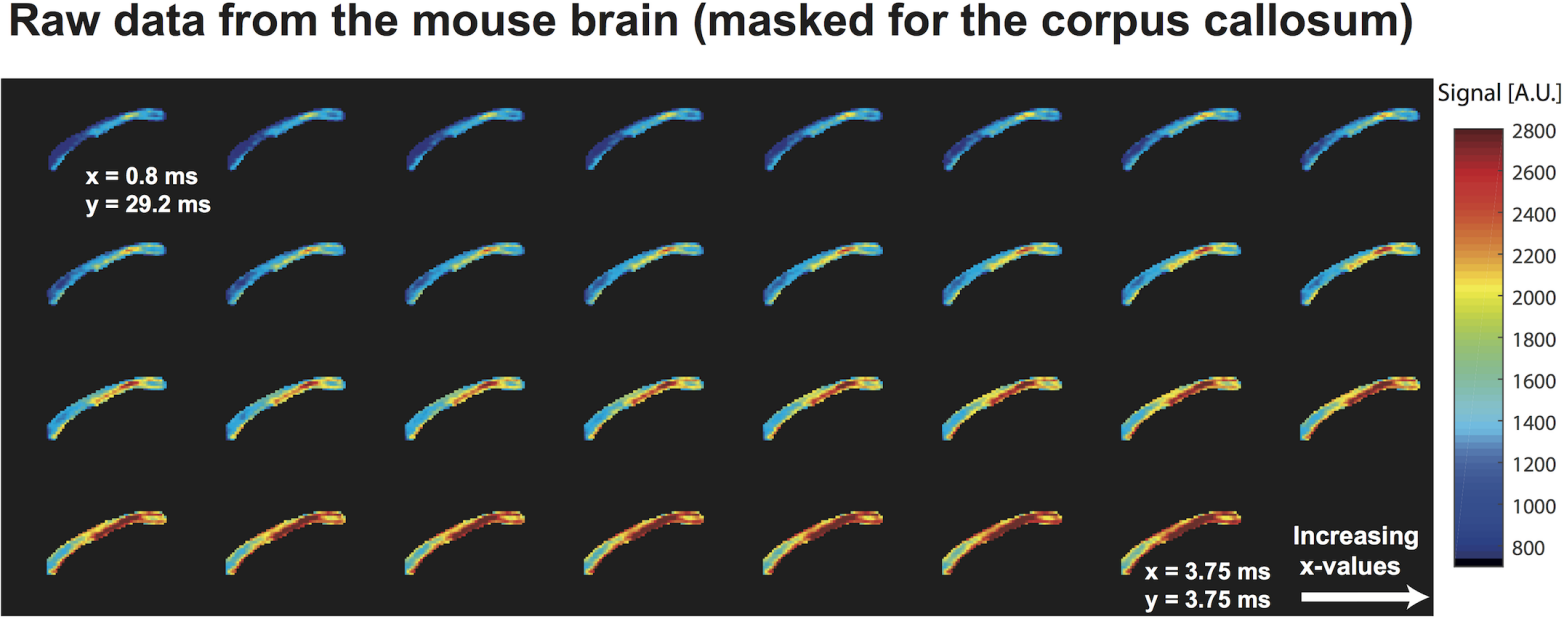
Raw NOGSE MRI data arising from sagittal images of a mouse brain, masked for the corpus callosum and plotted as a function of increasing *x-*values. Notice the clear increase in signal intensity with increasing *x*-values, as the weighting gradient transitions from a mostly long bipolar block to an OGSE-like sequence–while always retaining a constant-time fashion. Notice as well the different profiles evidenced by the various corpus callosum sub-sections. The diffusion gradients were along the phase-encoding direction, *i*.*e*. along the vertical axis of the image.

Given this potential to reconstruct size distributions from a single-variable (*x*) experiment, NOGSE was combined with a fast MRI protocol as shown in Fig 1A, and used to map an intact mouse brain. Brain’s WM in particular is often targeted in microstructural characterizations, given the importance of axonal sizes in defining conduction velocities [4,43], and their potential structural correlations with CNS diseases [7]. NOGSE MRI signals were therefore collected and examined; raw data arising from these experiments are presented in Fig 3 for the corpus callosum–a prominent white matter structure–and clearly show the expected signal increase with increasing *x*-values.

**Fig 4:**
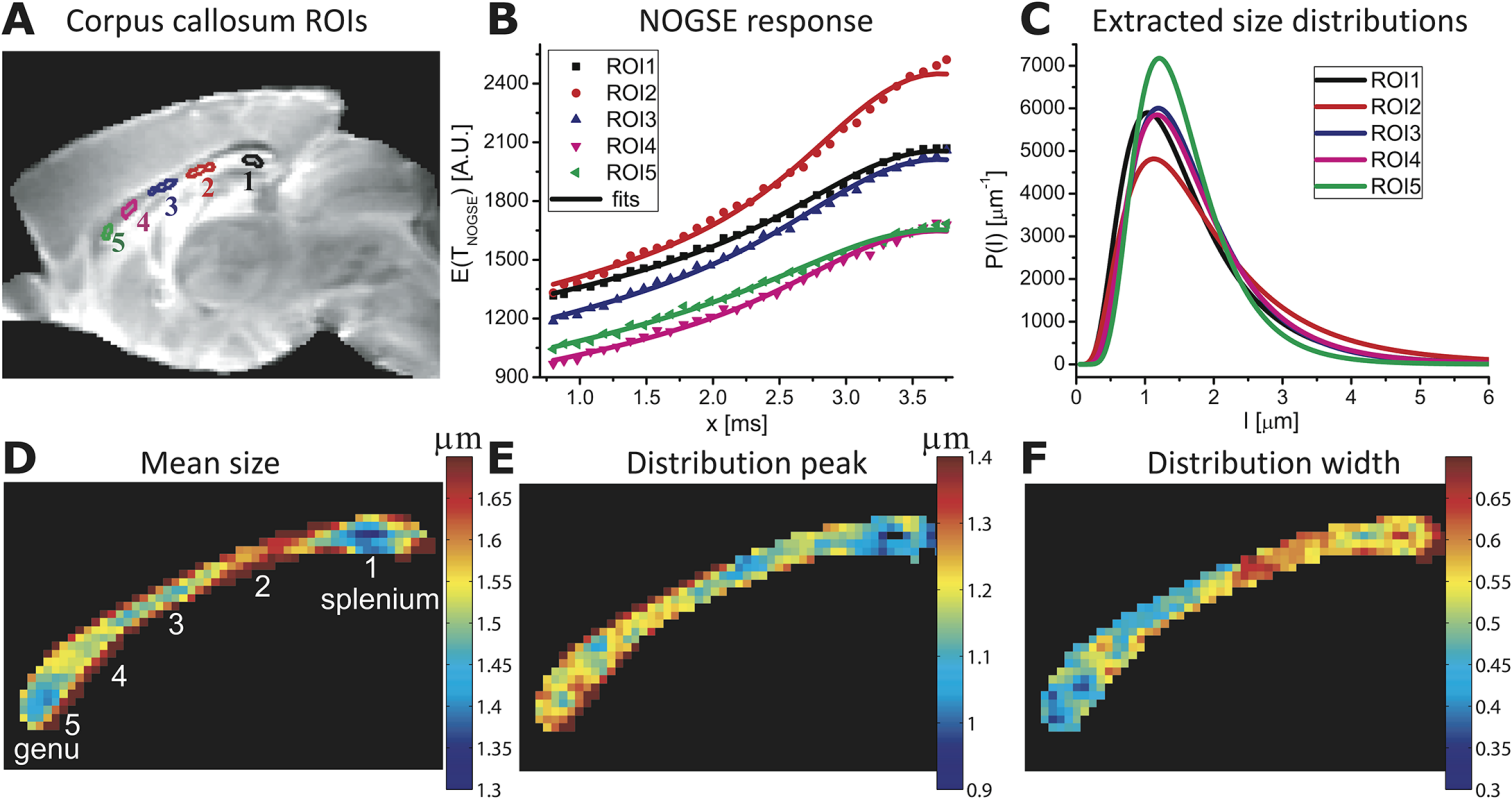
Mapping histological size distributions in a mouse corpus callosum. **(A)** Definitions of the various ROIs placed in different anatomical regions, superimposed on a reference MRI image. **(B)** Ensuing curves (symbols) and best fits (solid lines) arising from a NOGSE MRI experiment. **(C)** Size distributions *P(l)* extracted from (B), under a D_0_ = 0.7x10^-5^ cm^2^/sec assumption. **(D-F)** Maps of the mean, the peak and the width values extracted from pixel-by-pixel fits of the NOGSE response, highlighting the contrast between the corpus callosum different anatomical regions. NOGSE parameters: *T*_*NOGSE*_ = 30 ms, *N* = 8, *G*_*NOGSE*_ = 57.6 G/cm applied perpendicular to the main axis of the fibers in the corpus callosum (*i*.*e*., the vertical axis of the images). The extracted values describe the correlation lengths *l*_*c*_. See *Materials and Methods*for further parameters.

Fig 4 analyzes the corpus callosum data in closer detail. Fig 4B displays this structure’s NOGSE signals observed as a function of *x* for the five selected regions-of-interest (ROIs) defined in panel 4A, when the diffusion-sensitizing oscillating gradient is applied in a direction orthogonal to the main axis of the fibers (*i*.*e*., along the vertical axis for the displayed image planes). Each of these regions exhibits a slightly different *∆M*_*NOGSE*_ modulation; fits of these data to the size distributions’ lognormal parameters (Fig 4B, solid lines) led to the lognormal curves in Fig 4C, showing variations in the means, peaks and widths of the confinements for these various ROIs. The excellent agreement of the fitted curves with the experimental data is consistent with the lognormal-distribution assumption. Extending these analyses on a pixel-by-pixel fashion results in the compartment size maps shown in Fig 4D–4F. The mean size and the distribution width maps in particular demonstrate significant contrasts between different corpus callosum anatomical regions, providing a microstructure-based tissue segmentation. For instance, although the *genu* and *splenium* regions of the corpus callosum exhibit similar mean sizes, the width of their distributions appears larger in the latter–consistent with human-based histological results [44]. Furthermore, the corpus callosum midsection exhibits larger compartment sizes distributed with a larger width. Overall these results suggest five morphologically distinct regions (Fig 4D), in good agreement with hallmark anatomical segmentations observed in end-point histological human studies [44,45]. These NOGSE-derived maps also resemble those in a recent study that employed pseudo-2D q-space MRI and pixel clustering, to portray size distributions in a rat’s corpus callosum [46]. Notice, however, that the quantitative results summarized in Fig 4 are obtained in a 1D fashion, and do not need to invoke clustering or other models of the tissue’s microstructure.

**Fig 5:**
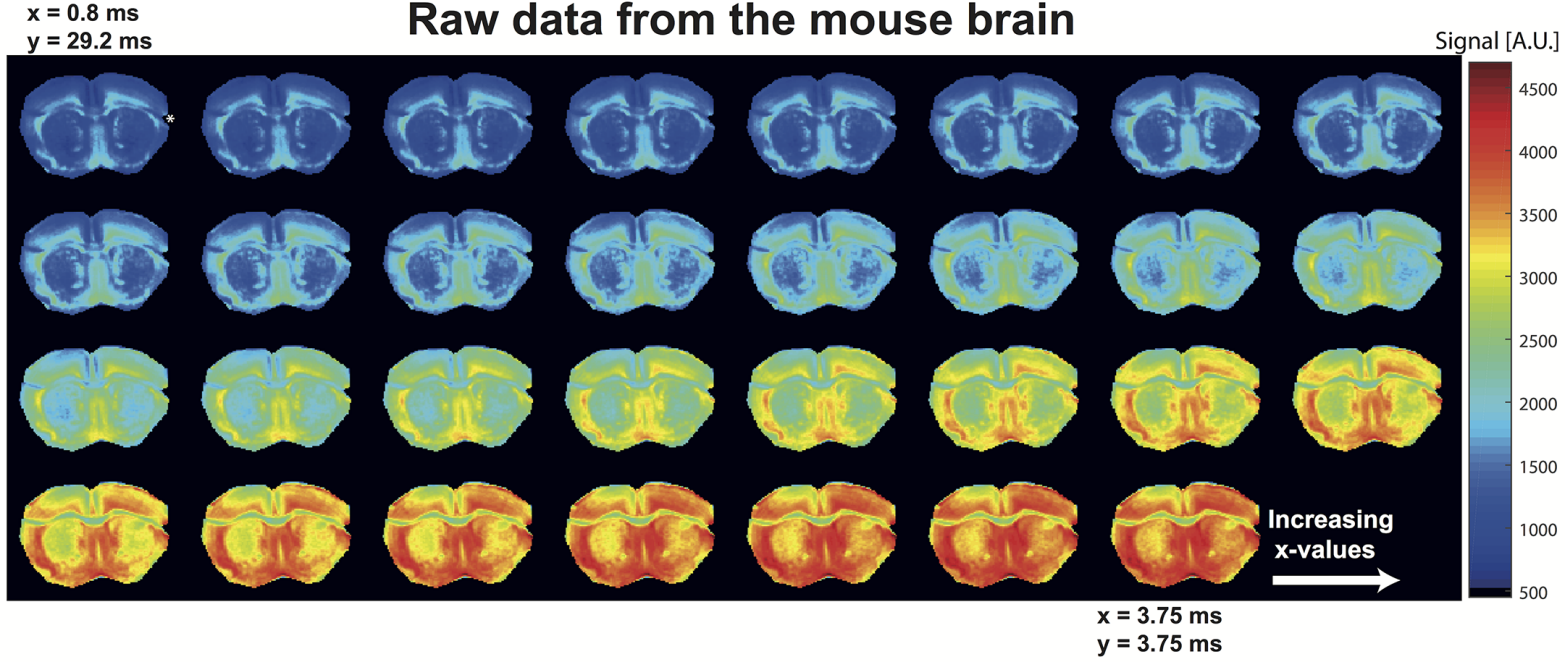
Idem as in Fig 3, but showing raw NOGSE MRI data from coronal images of a mouse brain. Different brain regions manifest different NOGSE signal increases with increasing *x*-values, even within the gray matter. These features allow for the microstructural segmentations shown in the main text. The asterisk in the top-leftmost image represents a tissue area damaged upon preparation.

**Fig 6:**
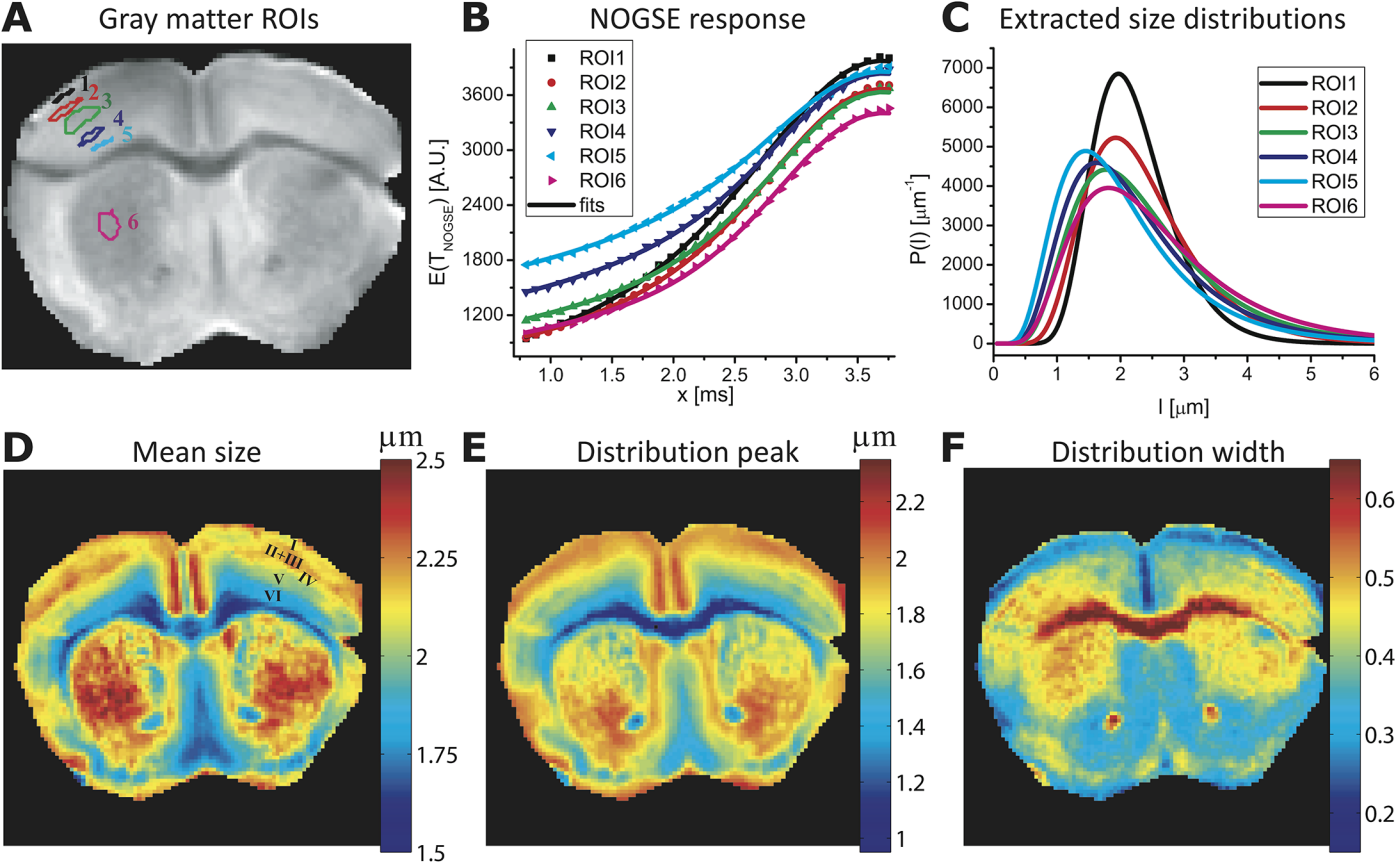
Mapping size distributions of a mouse’s gray matter. **(A)** ROI definitions of various GM regions. **(B)** NOGSE curves from these ROIs (symbols) along with the size distribution fittings in each ROI (solid lines). Note the stronger amplitude modulation in the gray matter compared with the WM shown in Fig 3. **(C)** Size distributions extracted from the data in (B). **(D-F)** Maps of mean sizes, peak values and distribution widths obtained by fitting the NOGSE data retrieved from a mouse brain, reflecting the correlation lengths *l*_*c*_. Cortical layering can be observed, and are marked with Roman numbers on the Mean size map. NOGSE parameters: *T*_*NOGSE*_ = 30 ms, *G* = 57.6 G/cm, *N* = 8; the gradient was oriented along the left-right axis of the image, and *D*_*0*_ was assumed 0.7x10^-5^ cm^2^/sec.

Due to their higher, more coherent cellular organizations, most microstructural diffusion MRI studies focus on white matter characterizations. Structural disorder at a cellular level has made studying microstructural features of gray matter (GM) much more challenging [47,48]. In view of this we sought to explore NOGSE’s size distribution contrast capabilities to identify GM’s most salient morphological feature: its cortical layering [49,50]. To this end a mouse brain was imaged along a coronal plane; Fig 5 shows raw data arising from such experiments, demonstrating once again a well-behaved and characteristic NOGSE response for all pixels, both in the grey and the white matter. ROIs were selected from these data in different cortical gray matter prominent features including the cortical layers and deep gray matter of the striatum (Fig 6A). The ensuing NOGSE signals, shown in Fig 6B, exhibit once again region-dependent responses. On comparing these with the responses in Fig 4B, NOGSE’s amplitude modulations appear larger in GM than in WM counterparts. This reflects the typically larger mean cellular sizes that characterize GM over WM axon counterparts, as evidenced upon comparing the distributions in Figs 4C and 6C. Note again the excellent agreement between the fits and data in Fig 6B, with *σ* and *l* (besides the overall amplitude of the signal) as the only free parameters to adjust; this consistency lends further support to the usefulness of the lognormal-distribution assumption. Pixel-by-pixel maps of the NOGSE-derived GM size distributions are shown in Fig 6D–6F, and point to several remarkable features. One concerns the markedly different contrasts that the mean, the width and the peak of the size distributions yield in the cortical GM. The contrast afforded by the mean sizes of these distributions closely resembles known features of the cortical layering. These layers, identified in Fig 6D as I, II+III, IV, V and VI, have thicknesses of ~ 90, 450, 180, 360 and 450 μm, respectively. These thicknesses are consistent with literature values for mice cortical brain layers [50]. Interestingly, the peak map exhibits a similar but not identical layering contrast: layers I, II and III appear to have similar distribution peaks (Fig 6E); layers are even further melded together in the distribution width map. This highlights the need for a sensitive technique capable of furnishing a full morphological characterization of size distributions, vis-à-vis other methods that may rely solely on one parameter (like the average compartment size) to deliver this information. It should be further noted that diffusion anisotropy effects–though typically quite small in gray matter [47]–would also need to be considered if proceeding with a further refinement of this model.

## Discussion

This study sought to test the feasibility of extracting cellular-scale size distributions parameters–including means, peaks and widths–using a microstructural sequence that like NOGSE, exhibits a $l_{c}^{6}$ dependence. Experiments show that such distributions can indeed be accurately characterized from simple curves involving a single variable and moderate gradient amplitudes. NOGSE’s robustness reflects the good contrast that small-sized structures like the ones targeted in this study endow it, a constant-time nature freeing it from T_2_-derived decays, and a constant number of gradient switchings that reduce potential sources of error. We further note that although NOGSE’s $l_{c}^{6}$ term may exist in principle also in OGSE, it has insofar not been used and would require special precautions to unambiguously reveal it.

When coupled to MRI’s non-invasive mapping abilities, NOGSE’s contrast opens a wealth of vistas for the *in vivo* characterization of tissue structures in general, and of the CNS in particular. These types of “virtual histology” characterizations could depict aberrations in cellular morphologies in a range of pathologies including de- and dys-myelination of axons [51] and changes in neural densities and sizes [52]–information which is usually evidenced only upon post-mortem histological evaluations. Noninvasively tracking the longitudinal evolutions of such size-distribution contrasts in the CNS via NOGSE MRI experiments could also open new vistas in understanding, at a physiological level, the evolution of cognitive and behavioral activities.

At an analytical level it is worth stressing that although this study focused on lognormal distributions, experimental findings do not have to fulfill this specific scattering to yield a meaningful insight. Ongoing studies are showing that NOGSE can also reveal other distributions, including multi-modal ones. Indeed, a strong point of the presented results is that the only “model” that it needs to invoke is that spins diffuse with a uniform coefficient *D*_*0*_ that disperses their evolution phases according to the Gaussian Phase Approximation [53]. One might argue that the assumption of a uniform *D*_*0*_, particularly in the brain, may compromise our results; however, when *D*_*0*_ was mapped via very rapid gradient oscillations and raw data like those shown in Fig 6 were refitted point-by-point to account for pixel-specific diffusivities, no significant differences in the derived distributions could be observed. This robustness can be ascribed to NOGSE’s exponential contrast, which varies as the relevant lengths to the power of six but only as $D_{o}^{-2}$: variations in *D*_*0*_ will therefore shift slightly the absolute values of the extracted distributions, yet their intrinsic contrast will be preserved.

One may also wonder whether the Lorentzian approximation adopted here for the spectral density function, is an optimal one. Whereas this assumption is often adopted for completely hermetic pores [27,39], its validity may be compromised if restrictions are incomplete, in the presence of multi-compartment exchange processes, or if mixed diffusion modes are considered. Moreover, the assumption of a lognormal distribution of pore sizes implies that each pore is independent, and as such it constrains the possible complexity of the entire system, whose pores could be interconnected leading to different types of diffusion processes. An alternative approach could have consisted of assuming a more complex *S*(*ω*) form, that includes these effects. Still, given the very good agreement observed between experimental results and the simpler Lorentzian model, this research did not justify resorting to more complex spectral density forms: data could be fitted well by suitable Lorentzian distribution parameters. Still, further theoretical, computational and experimental studies are needed to determine whether this distribution is the most adequate characterization to describe spectral densities in more complex pore structures.

It should also be noted that the $l_{c}^{6}$ parametric-sensitivity is not an automatic guarantee of better size distribution characterizations. Indeed, in unfavorable instances, the applied gradients may be too strong or the pores too big, leading to the erasing of contributions from larger structures and/or data that lack sufficient signal sensitivity. Still, in the context of CNS-relevant pores, one is often interested in the smaller (<10 μm) dimensions, where NOGSE’s *l*_*c*_^*6*^-dependence should be beneficial for practical gradient values. Another point worth highlighting is that NOGSE experiments, at least as here performed, are not rotationally invariant; hence, the corpus callosum analyses require that the gradients be applied perpendicular to the main axis of the fiber. Nevertheless NOGSE experiments could be performed in alternative ways; and it would be interesting to explore whether some of these options could lead to rotationally invariant metrics. One should also notice that maximizing NOGSE’s contrast will come at the expense of prolonging the TE, an attribute that may bias NOGSE towards a tissue’s longer T_2_ species. Still, at preclinical and clinical fields this limitation should not be too restricting. Furthermore, one could envision performing NOGSE MR Spectroscopy on CNS metabolites which have very long T_2_s, to endow the measurement with enhanced specificity towards intra / extra cellular compartments. Double-NOGSE modes for probing the eccentricities of orientationally-dispersed morphological distributions [47,54], can also be envisioned. Last but not least, the NOGSE waveforms could be split around the refocusing pulse, thereby garnering greater immunity towards sources of artifacts such as eddy currents, or internal susceptibility-induced gradients.

**Fig 7:**
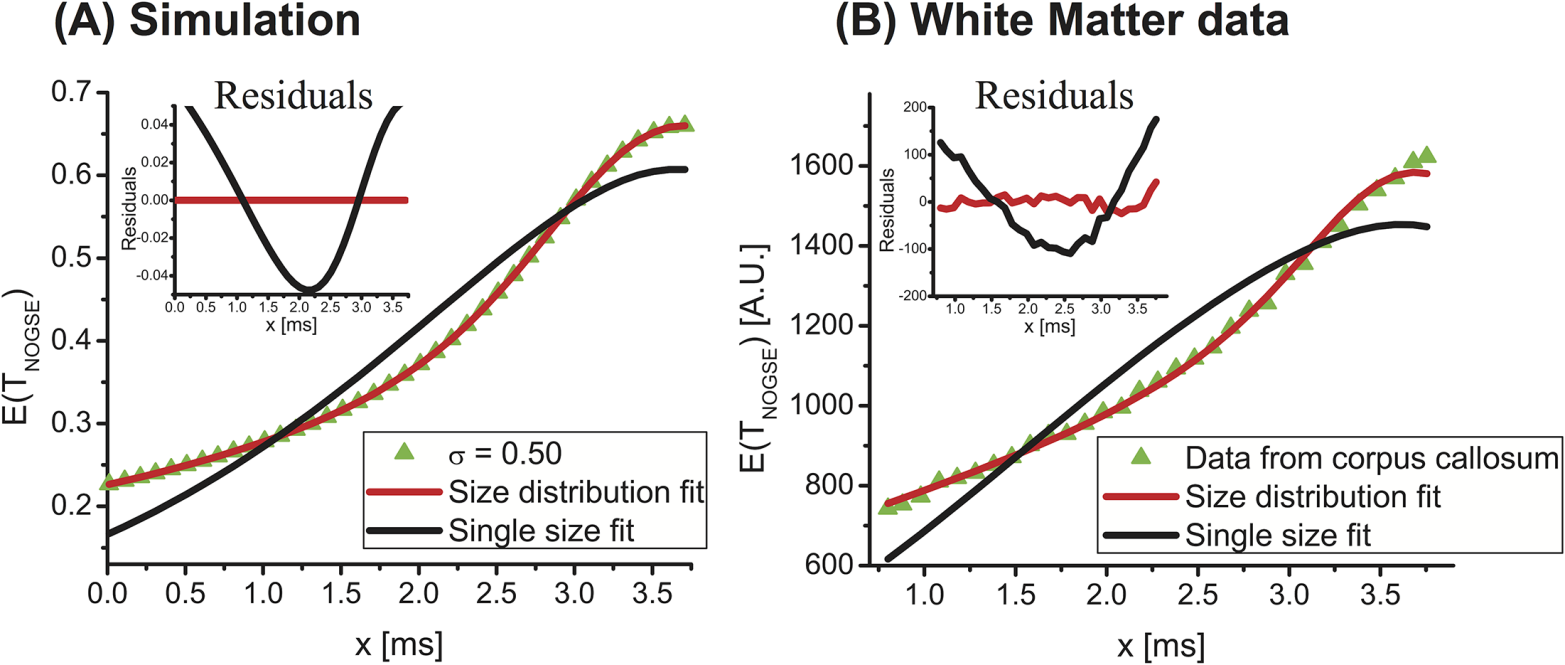
On the need for distributions to describe the NOGSE response arising in brain tissues. **(A)** Simulation for NOGSE data arising from a distribution characterized by *l*_*c*_ = 2 μm and σ = 0.5 at *G = 57*.*6* G/cm, *N = 8*, and *T*_*NOGSE*_ = 30 *ms* along with fits to the distribution (red curve) and an attempt to fit just a single size to the data (black curve). Residuals of the fits are shown in the inset. **(B)** Idem but for experimental data arising from ROI #4 of the corpus callosum (see Fig 4A for the ROI’s definition). The residuals clearly demonstrate the need for distributions to fit the data in a robust way.

Another feature worth highlighting is the actual need to introduce size distributions, to accurately describe the NOGSE responses arising from the brain. Fig 7A stresses this with a synthetic NOGSE data arising from a modest lognormal distribution (green symbols), along with fits of these data to a single size (black curve) and to a distribution (red). Clearly, the data demands being fitted by a distribution in order to reach reasonable residuals. Fig 7B demonstrates this experimentally with a representative ROI taken from the mouse brain WM. The excellent agreement then resulting between the experimental data and the fits by adding just a single extra parameter to the model, lends support to the need for using a distribution of sizes like the one derived from the lognormal shapes hereby used to describe the experimental data. Notice as well the similarity between the behavior of the residuals in both the synthetic and experimental data panels. Remarkably, similar trends were observed for *all* the ROIs examined in this mouse brain study, both in the gray and white matter regions. While this does not unequivocally prove that the experimental deviation from a single compartment model is due to the presence of size distributions, it lends strong support to the hypothesis that these distributions effects are indeed observed. These microstructural findings bode well for future characterizations of size distributions in the contexts of health, function and disease. Further studies will be required to test the stability of these results among larger cohorts of brains, incorporating end-point histology to quantitatively correlate the NOGSE observations with axonal diameter distributions. Furthermore, following the overall framework given here, it would be interesting to test whether the contributions of different tissue components (e.g., intra/extra-cellular environments) and geometries (e.g., packing, dispersion, etc.) could be modeled, and their effects included into this kind of studies. In this sense, the data from the cortex (Figs 5 and 6) are particularly interesting. Although diffusion anisotropy (as obtained from, e.g., DTI), is generally low in the cortex as well as in most other gray matter areas of the brain, the gray matter’s underlying microstructures are indeed highly heterogeneous [47,48]. The NOGSE experiment seems to nevertheless show prominent features of cortex; this could be a result of its filtering of the larger components (such as cell bodies), and from having an enhanced sensitivity towards the smaller sizes (as manifest in the ~2–3 μm compartments denoted in Fig 6). Also, as axons project radially in the cortex, it is plausible that NOGSE tracks to some extent the orientation of these fibers. Future studies will focus on deriving rotationally invariant metrics from NOGSE, an interesting vista as these distribution parameters could become a novel way of characterizing orientations in the brain.

**Fig 8:**
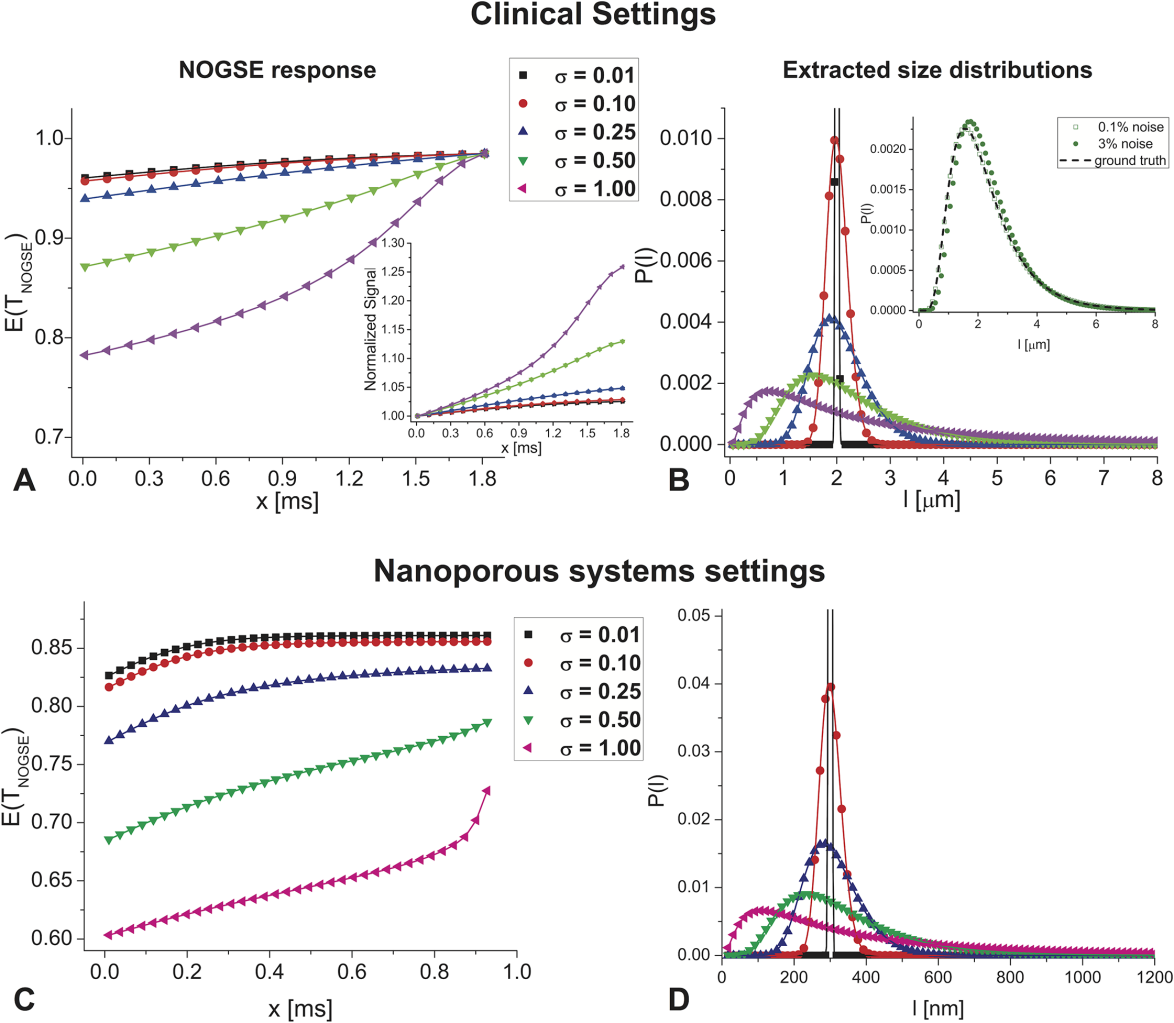
NOGSE’s size-resolving potential in human- and materials-oriented setting. **(A-B)** Simulations predicting NOGSE’s ability to extract cellular size distributions in clinically-relevant settings, involving *G =* 6 G/cm, *N =* 64, and *T*_*NOGSE*_ *=* 120 ms, *D*_*0*_ = 3.0E-5 (cm)^2^/sec. Notice that even when assuming the relatively weak gradients available in whole body MRIs, cell-sized distributions can be resolved and characterized. The inset in panel B analyzes the effect of 0.1 and 3% noise added to the third distribution, showing that with some noise levels, distribution can still be reconstructed. All definitions are akin to those in Fig 1B and 1C. **(C-D)** Simulations demonstrating NOGSE’s ability to extract pore distributions in mesoporous materials (10–1000 nm range), using the stronger diffusion gradients available in NMR scanners (*G =* 200 G/cm, *N =* 160, and *T*_*NOGSE*_ *=* 150 ms, *D*_*0*_ = 0.41E-5 (cm)^2^/sec). Notice the strong differences in signals arising when pores are distributed around *l*_*c*_ = 300nm.

It is worth concluding with a reflection on the potential relevance of this study’s experiments for human investigations on the one hand, and for characterization of porous media in general on the other. The cellular-level characterizations in Figs 4 and 6 were performed using NOGSE gradient peak amplitudes of 57.6 G/cm; although this is a reasonable gradient strength for systems designed to investigate animal models, it is significantly higher than gradient strengths available in clinical scanners. Still, since NOGSE’s amplitude modulation varies as *ΔM*_*NOGSE*_ = $\frac{1}{4{\text{D}}_{0}^{2}}\;{\left(\gamma \text{G}\right)}^{2}\;*\;\left(\text{N}-1\right)\;{\text{l}}_{\text{c}}^{6}$, NOGSE’s parametric-sensitivity towards compartment sizes can be increased not only by raising *G*, but also with *N* [34]. At the lower fields used in clinical studies T_2_ is substantially increased; *T*_*NOGSE*_ can therefore be made significantly longer than the 30 ms values used in this study, and thereby accommodate more *N* oscillations for the same range of *x*-values as were used here. Although the “dynamic-range” of the amplitude modulations may be reduced (a result of trading the square dependence on *G* for a linear dependence on *N*), simulations akin to those shown in Fig 1 for clinically-relevant parameters (*G =* 6 G/cm, *N* = 64, *T*_*NOGSE*_ = 120 ms, *D*_*0*_ *=* 3^.^10^−9^ m^2^/sec) reveal that at least some cellular-sized distributions can be faithfully reconstructed, particularly if they have a slightly larger width (Fig 8A and 8B). Still, it is important to note that the smaller NOGSE’s amplitude modulation becomes, the more challenging it is to resolve narrower distributions. While these simulations rely on somewhat high *D*_*0*_ values–which could reflect diffusivity in white matter [55]–as well as somewhat higher gradient amplitudes than are conventionally present (e.g., 4 G/cm), we note that if a more conventional *D*_*0*_ value of 1.5^.^10^−9^ m^2^/sec along with a more conventional clinical gradient amplitude are chosen, NOGSE’s *G* ^*2*^ */D*_*0*_^*2*^ dependence would render it even more sensitive, with its amplitude increasing over these calculations by a factor of ~80%. In this respect, a new generation of scanners possessing stronger gradients, could be particularly useful to enhance the quality of such experiments. A second realm where NOGSE-based size distribution characterization methods could apply, arises in the realm of mesoporous materials. These are important catalytic systems where relevant pore sizes vary between a few nanometers and fractions of microns [56]. Fig 8C and 8D show the expected NOGSE-derived distributions arising from such media, assuming pores distributed around a typical size of 300 nm. While these sizes are over an order of magnitude smaller than those involved in tissue studies, NOGSE still demands gradient strengths that are normally available on contemporary microimaging NMR scanners, to reconstruct these pore size distributions. Even pore-size distributions in the neighborhood of 100 nm can be characterized in this way, reflecting the relevance of NOGSE to enable these MR-challenging characterizations vis-à-vis pore size distributions.

In summary, this study presented a new approach to unravel cellular size distributions using a simple experiment monitoring the time-dependent transitions between free and restricted diffusion regimes. Simulations and *in-cell* validations demonstrate the reliability of the approach; when combined with MRI-based mapping techniques, remarkable contrasts demonstrating structures and size distributions consistent with *ex vivo* histological analyses, were evidenced in both white and grey matter tissues. All these features augur well for further exploiting this approach in novel characterizations of microstructures in porous systems in general, and for studying microstructural correlations of normal and diseased CNS in particular.

## Supporting Information

S1 Fig NOGSE simulations for distributions centered around different correlation lengths. The left panels shows NOGSE signals expected for *l*_*c*_ = 1 and 4 μm, for the different distributions indicated by the symbols. The right panels show the corresponding size distributions extracted (symbols) along with the ground truth (lines) by fits of the NOGSE data. The lines in the left panel are then fits generated from simulating NOGSE signals, from the distributions reconstructed from the right-panel fits. Simulation parameters: *G* = 40 G/cm, *T*_*NOGSE*_ = 30 ms, *N* = 8, *D*_*0*_ = 0.7^.^10^−5^ cm^2^/sec.
(PDF)

S2 Fig Analysis of the NOGSE signals in Figs 4 and 6, as a function of a *b* value. The *b* value of the gradient modulation waveform is defined as [(*N*-1)(*x*/*T*_NOGSE_)^3^+(1-(*N*-1)(*x*/*T*_NOGSE_))^3^] *G* ^2^ (*T*_NOGSE_)^3^/12. The figures clearly show the non-exponential behavior manifesting the restriction effects of the diffusion process.
(PDF)
