# Supplementary figures and images for: Size Distribution Imaging by Non-Uniform Oscillating-Gradient Spin Echo (NOGSE) MRI
Source: PLoS One. 2015 Jul 21;10(7):e0133201. doi: 10.1371/journal.pone.0133201 (PMC4509907; doi:10.1371/journal.pone.0133201)

$l_c = 1 \mu\text{m}$

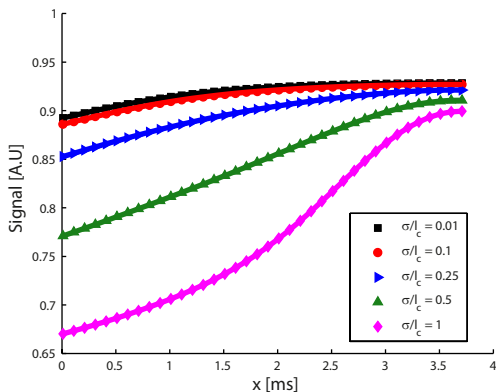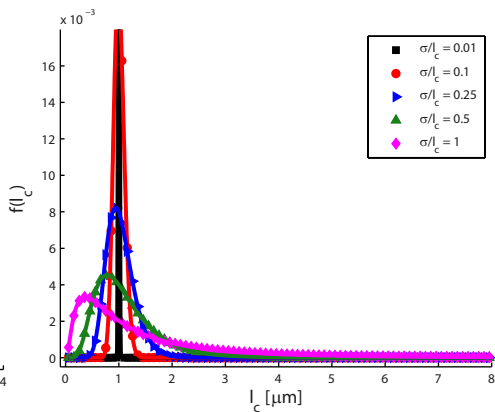

$l_c = 4 \mu\text{m}$

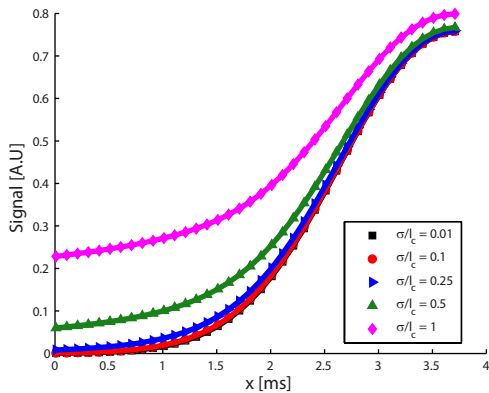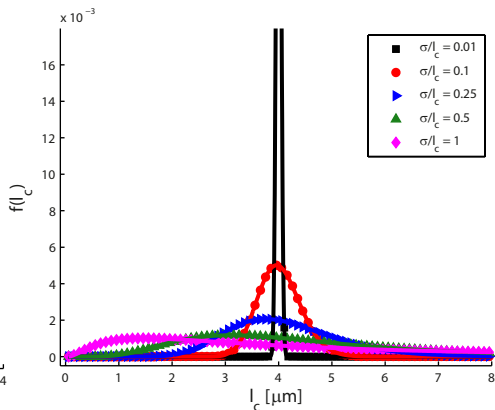

Supplement: S1 Fig — The left panels shows NOGSE signals expected for l c = 1 and 4 μm, for the different distributions indicated by the symbols. The right panels show the corresponding size distributions extracted (symbols) along with the ground truth (lines) by fits of the NOGSE data. The lines in the left panel are then fits generated from simulating NOGSE signals, from the distributions reconstructed from the right-panel fits. Simulation parameters: G = 40 G/cm, T NOGSE = 30 ms, N = 8, D 0 = 0.7.10−5 cm2/sec. (PDF) [file pone.0133201.s001.pdf]

Corpus callosum ROIs

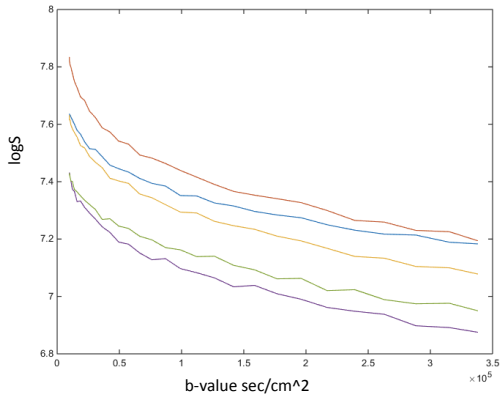

Coronal ROIs

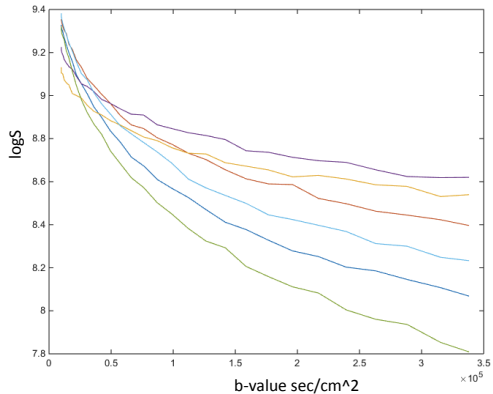

Supplement: S2 Fig — The b value of the gradient modulation waveform is defined as [(N-1)(x/T NOGSE)3+(1-(N-1)(x/T NOGSE))3] G 2 (T NOGSE)3/12. The figures clearly show the non-exponential behavior manifesting the restriction effects of the diffusion process. (PDF) [file pone.0133201.s002.pdf]
